# Toward the accurate estimation of elliptical side orifice discharge coefficient applying two rigorous kernel-based data-intelligence paradigms

**DOI:** 10.1038/s41598-021-99166-3

**Published:** 2021-10-05

**Authors:** Masoud Karbasi, Mehdi Jamei, Iman Ahmadianfar, Amin Asadi

**Affiliations:** 1 Water Engineering Department, Faculty of Agriculture, University of Zanjan, Zanjan, Iran; 2 Faculty of Engineering, Shohadaye Hoveizeh Campus of Technology, Shahid Chamran University of Ahvaz, Dasht-e Azadegan, Susangerd, Iran; 3 Department of Civil Engineering, Behbahan Khatam Alanbia University of Technology, Behbahan, Iran; 4 Institute of Research and Development, Duy Tan University, Da Nang, 550000 Vietnam; 5 Faculty of Natural Sciences, Duy Tan University, Da Nang, 550000 Vietnam

**Keywords:** Computational science, Mechanical engineering

## Abstract

In the present study, two kernel-based data-intelligence paradigms, namely, Gaussian Process Regression (GPR) and Kernel Extreme Learning Machine (KELM) along with Generalized Regression Neural Network (GRNN) and Response Surface Methodology (RSM), as the validated schemes, employed to precisely estimate the elliptical side orifice discharge coefficient in rectangular channels. A total of 588 laboratory data in various geometric and hydraulic conditions were used to develop the models. The discharge coefficient was considered as a function of five dimensionless hydraulically and geometrical variables. The results showed that the machine learning models used in this study had shown good performance compared to the regression-based relationships. Comparison between machine learning models showed that GPR (RMSE = 0.0081, R = 0.958, MAPE = 1.3242) and KELM (RMSE = 0.0082, R = 0.9564, MAPE = 1.3499) models provide higher accuracy. Base on the RSM model, a new practical equation was developed to predict the discharge coefficient. Also, the sensitivity analysis of the input parameters showed that the main channel width to orifice height ratio (B/b) has the most significant effect on determining the discharge coefficient. The leveraged approach was applied to identify outlier data and applicability domain.

## Introduction

Water diversion structures comprised of side weirs, side intakes, side orifices, and sluice gates are the most crucial and widespread devices in sewerage, irrigation, and agricultural systems which controllably divert and transfer the flowing water from the main channel to a tributary channel^1^. The flow regime in water diversion devices is categorized into the spatially varied flow with decreasing flow discharge^2,3^. In the past four decades, numerous investigations have been conducted on hydraulic characteristics side structures, including side wires^1,4–8^, and Side Sluice Gate^9–12^.

As one of the most important diversion structures, the side orifices are usually placed on the side of the open channel, at a specific height from the bed, to distribute the flow discharge to outside the channel in the aim of irrigation and drainage systems and wastewater treatment plants^13^. The importance of measuring the lateral flow through the orifices in hydraulic systems has led to considerable research efforts devoted to laboratory investigations on various shapes of side orifice in open channels^14^. Ramamurthy et al.^15^, as a pioneer researcher, studied the flow mechanism through a rectangular lateral orifice in a rectangular open channel. Besides, they theoretically and experimentally analyzed the characteristics of flow through the lateral weir-orifice unit^16^. Gill^17^ and Ojha and Subbaiah^18^ presented the theoretical analysis of flow characteristics through various side slots in free-surface and pressure flow conditions.

Moreover, Swamee^19^ studied discharge coefficient estimation for flow through weir-orifice units under free-flow conditions. Recently, Hussain et al.^20^ experimentally and theoretically accomplished the sharp-crested circular side orifices in open rectangular channels under free-flow conditions. Besides, they extended their investigation on the lateral circular orifice under both free and submerged flow conditions. They derived various discharge coefficient relationships with acceptable accuracy, which usually were depended on upstream hydraulic characteristics^21^. Also, Hussain et al.^22^ reported an analytical and laboratory study on the hydraulic characteristics of flow-through side square orifices in rectangular open channels. They provided a new discharge coefficient relationship based on the approach flow Froude number and ratio of orifice and channel width. Hussain et al.^23^ conducted extensive laboratory and theoretical research on the performance of sharp-crested rectangular side orifice under the free-flow condition compared to square and circular side orifice. They found that the circular orifice is more efficient to divert flow than square side orifice by the same opening area. Besides, they developed their research in the aim of modifying the concept used by Ramamurthy^15^ in the derivation of discharge coefficient relationship for flow through lateral side rectangular orifice.

More recently, Vatankhah and Mirnia^13^ conducted an experimental and analytical study to predict the discharge coefficient of sharp-crested side triangular orifices based on 750 laboratory experiments under free-flow conditions. They proposed different discharge coefficient relationships for two scenarios: the approach Froude number and the absence of one. Furthermore, Vatankhah and Rafeifar^3^ assessed the operation of the elliptical sharp-crested side orifices for diverting flow from the horizontal open channel. This research examined both small and large elliptical side orifices for deriving the regression-based discharge coefficient relationships in two scenarios depending on Froude's approach and without it. In the current research, it should be mentioned that 588 laboratory experiment tests for data-driven based assessment of elliptical side orifice discharge coefficient.

Artificial intelligence and machine learning models in different engineering problems such as hydraulic^24–26^, geotechnical^27–31^, and mechanical^32^ engineerings have become very popular. In two recent decades, laboratory equipment and human errors, on the one hand, the complexity and nonlinear behavior of spatially varied flow through these facilities and the insufficient accuracy of classical regression-based methods, on the other hand, has caused several researchers to turn their attention to the data-driven and machine learning techniques^33–36^. Numerous attempts focus on the application of conventional artificial intelligence (AI), such as an artificial neural network (ANN) and adaptive neuro-fuzzy inference system (ANFIS), to measure the coefficient of discharge through the side lateral weirs and side orifices. For instance, the mentioned approaches implemented to predict the discharge coefficient for rectangular sharp-crested weirs^37–40^; for measuring the discharge capacity of rectangular sharp-crested side weirs in sewer systems^41^; to assess the discharge coefficient of triangular and trapezoidal labyrinth side weirs; for estimating the discharge coefficient for a semi-elliptical labyrinth side weirs^42^; to accurate determination of the discharge coefficient for a triangular side weir under subcritical flow conduction^26,43^, and predict the discharge of rectangular and circular side orifices in a rectangular channel^44^. The bedside, Gene expression programming (GEP) paradigm has been employed to determine the discharge coefficient of rectangular side weirs in various flow regimes along the rectangular and trapezoidal channels^45,46^. In other cases, a support vector machine (SVM) model was employed to model the discharge coefficient of a side weir in a rectangular^47^ and trapezoidal channel^48^; multivariate adaptive regression splines (MARS) and the group method of data handling (GMDH) have been utilized to predict the discharge coefficient of Weir-Gat^49^.

Furthermore, More recently, the self-adaptive extreme learning machine (SAELM) as a novel ML approach has been employed to model the side weirs discharge on converging channels^50^ and circular and rectangular side orifices along the open channel^51–53^. Jamei et al. applied three data-driven approaches, multiple linear regression with interaction (MLRI), locally weighted learning regression (LWLR), and multiple linear regression (MLR), to estimate the discharge coefficient of a triangular side orifice. Their results demonstrated the high capability of LWLR and MLRI models to estimate discharge coefficient^54^.

Literature review inferred that different ML-based methods had been used to model the discharge coefficient of hydraulic diversion devices. However, some structures have the complexity of hydraulic characteristics due to their particular shape. The accurate estimation of the discharge coefficient in them requires applying robust and efficient data-driven approaches.

In this research, the estimation of discharge coefficient in elliptical side orifice under free flow conduction, for the first time, has been assessed using two kernel-based data-intelligence paradigms, namely, Gaussian process regression (GPR) and kernel extreme learning method (KELM). Here, the response surface methodology (RSM), generalized regression neural network (GRNN), and empirical methods were adopted to validate the provided schemes. To the best of our knowledge, the proposed data-driven approaches have not yet been used for discharge measuring the hydraulics structures. Furthermore, the applicability domains of the provided paradigms were examined using the leverage approach, and a sensitivity analysis was performed to identify the most influential variables. Models were evaluated based on statistical indices, and the results were presented as tables and figures. This paper describes the experimental data and machine learning models in the second part (materials and methods). Results and discussion are presented in the third part, and at last, the conclusions are offered.

### Need for research

Determining the lateral flow in the side orifices is essential for water management, water pricing, and environmental engineering objectives. In the present study, to increase the accuracy of flow determination, using machine learning methods, GPR, KELM, GRNN, and RSM, models and relationships were developed to determine the flow coefficient of the elliptical side orifice.

## Material and methods

### Dimensional analysis and data preparation

**Figure 1:**
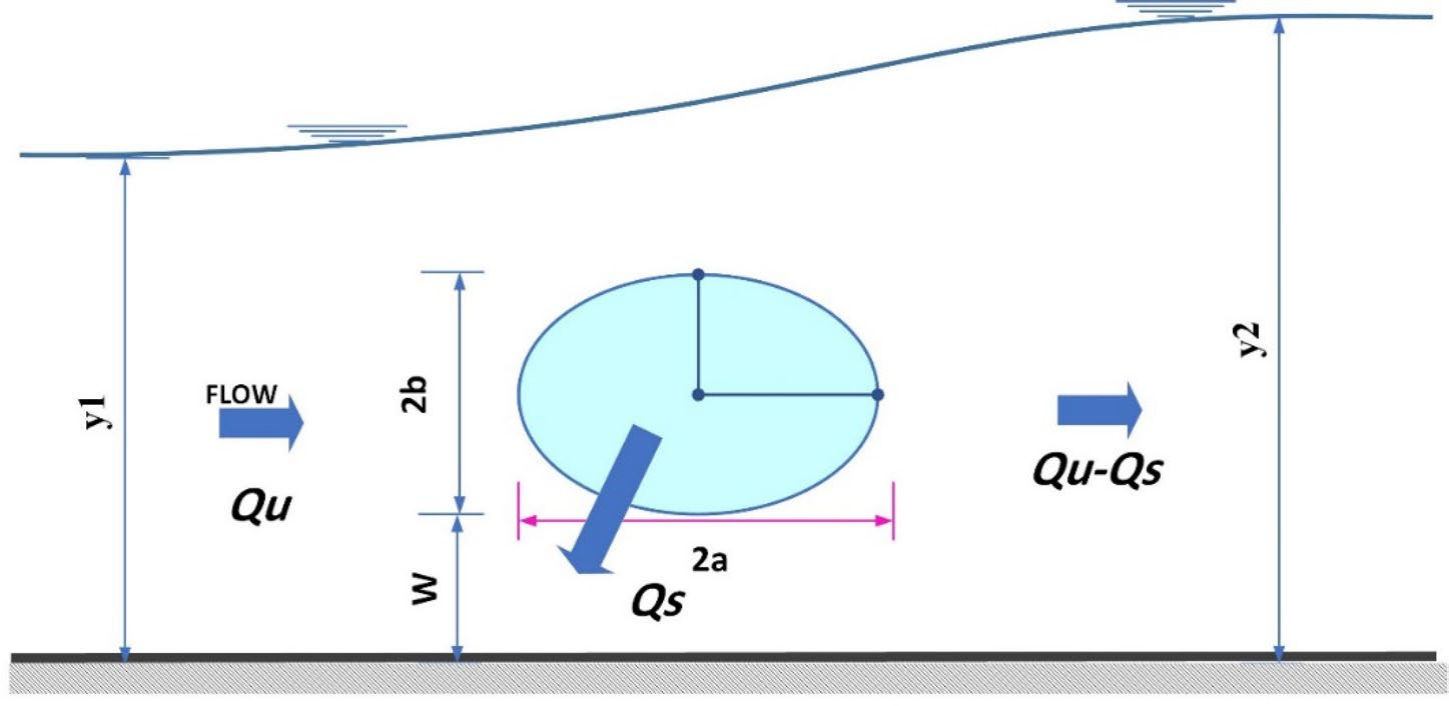
Schematic view of elliptic side orifice and its geometrical parameters.

Factors affecting the elliptical side orifices are (1) Dimension of the elliptic orifice ($a$ is horizontal semi-axis and $b$ is vertical semi-axis) (2) orifice crest height (the distance between the channel bed and the orifice) ($W$) (3) velocity in the main channel ($V1$) which is calculated as $V1 = Q/A_{1}$ (4) orifice upstream (*y*1) and downstream ($y2$) depths (5) Main channel width ($B$) (6) Gravity acceleration ($g$) (7) Water surface tension ($\sigma$) (8) Water dynamic viscosity ($\mu$) and (9) Water density ($\rho$)^3^. Figure 1 shows an elliptical side orifice and the geometric parameters used in it. According to the variables affecting the discharge coefficient of elliptical side orifice, a relation can be written as follows:

1 $$Cd = f1\left( {a,b,W,B,y2,V1,g,\rho ,\sigma ,\mu } \right).$$

Using Buckingham’s π theory, effective dimensionless parameters can be obtained as follows:

2 $$Cd = f2\left( {\frac{W}{b},\frac{B}{a},\frac{B}{b},\frac{y1}{b},Fr1 = \frac{V1}{{\sqrt {g \cdot y1} }},Re = \frac{\rho \cdot V1 \cdot a}{\mu },We = \frac{\sigma }{{\rho ,g \cdot a^{2} }}} \right).$$

Given that in open channels, the most critical effective force is gravity, the effect of viscosity and surface tension can be ignored^5,55^, so Reynolds and Weber numbers can be removed from the above equation.

3 $$Cd = f3\left( {\frac{W}{b},\frac{B}{a},\frac{B}{b},\frac{y1}{b},Fr1 = \frac{V1}{{\sqrt {g \cdot y1} }}} \right)$$

In the present study, Vatankhah and Rafeifar^3^ laboratory data, which includes 588 series of data, were used. They studied the effect of different geometrical and hydraulic parameters on the elliptical side orifice discharge coefficient. A horizontal rectangular channel (12 m length, 0.25 m width, and 0.5 m height) was used to perform experiments. Two types of rectangular and triangular weirs were used to measure the flow through the orifice (Qs) orifice and the upstream flow of the orifice (Qu). Two different lengths of orifice (a = 15, 20 cm), three heights (b = 2,3,4 cm) and 2 crest heights (w = 5, 10 cm) were used. In total, 12 different geometric shapes were created. (Qu) values ranged from 13.8 to 39.6 l/s, Qs ranged from 3.66 to 21.41 l/s, and the Froude number in the main channel ranged from 0.22 to 0.77. Finally, the discharge coefficient can be calculated as $C_{d} = Q/\pi ab\sqrt {2gh_{c} }$ where $h_{c} = y_{1} - W - b$. 588 laboratory data were randomly divided into two parts: training (75%) and test (25%). Table 1 shows the statistical specifications of training and test datasets.

**Table 1:** Statistical specifications of train and test datasets.

Data	Statistic	$W/b$	$B/a$	$B/b$	$b/y1$	$Fr1$	$C_{d}$
Train data	Mean	2.665	1.463	9.047	0.172	0.484	0.518
	Std	1.212	0.208	2.611	0.039	0.121	0.026
	Min	1.25	1.25	6.25	0.093	0.219	0.405
	Max	5	1.667	12.5	0.252	0.777	0.569
Test data	Mean	2.837	1.446	8.971	0.168	0.472	0.517
	Std	1.246	0.209	2.576	0.039	0.108	0.027
	Min	1.25	1.25	6.25	0.093	0.273	0.426
	Max	5	1.667	12.5	0.256	0.731	0.562

**Figure 2:**
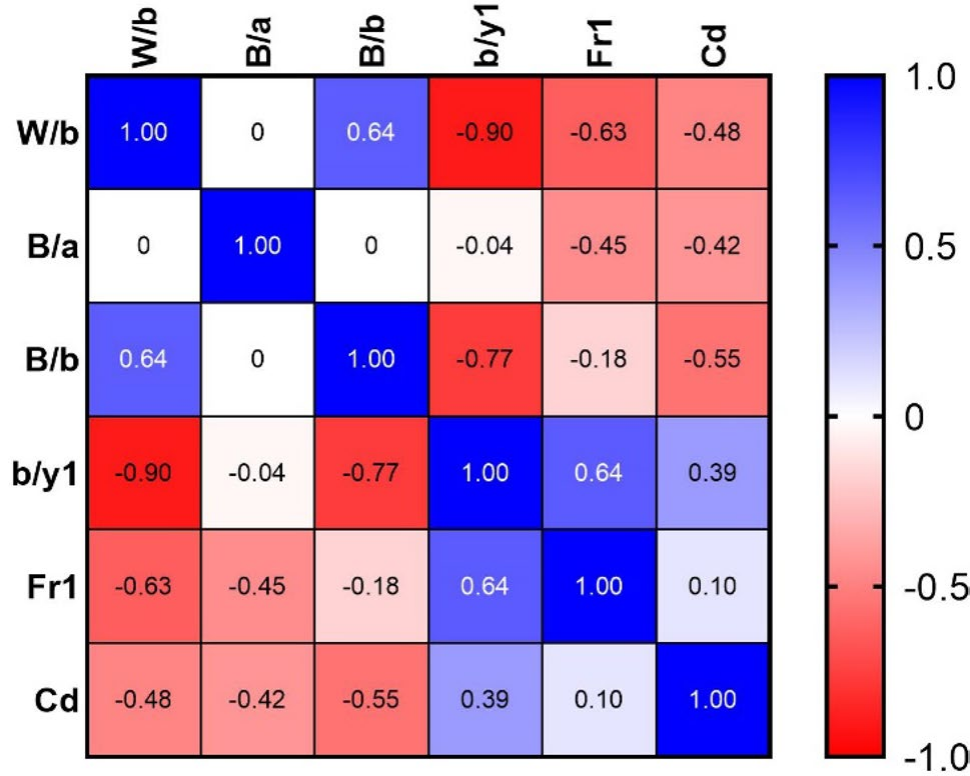
Correlation matrix of input(independent) variables and output (dependent) variable.

Figure 2 shows the relationship between the output variable ($$Cd$$) and independent variables for the dataset used in this study. The numbers in the figure represent the linear relationship between variables using the Pearson correlation coefficient. The value of this coefficient varies from −1 to 1. Positive values indicate a direct connection between variables, and negative values indicate an inverse relationship between variables. According to Fig. 2, the two variables $$b/y1$$ ($$r_{p} = 0.39)$$ and $$Fr1$$ ($$r_{p} = 0.10)$$ directly affects the discharge coefficient, which means that as they increase, the discharge coefficient increases. The three variables $$W/b$$ ($$r_{p} = - 0.48)$$, $$B/a$$ ($$r_{p} = - 0.42)$$ and $$B/b$$ ($$r_{p} = - 0.55)$$ have the inverse effect on the discharge coefficient, and as they increase, the discharge coefficient decreases. According to Fig. 2, the variables $$B/b$$ and $$W/b$$ have the highest absolute correlation with the elliptical side orifice discharge coefficient.

### Machine learning technics

#### Gaussian process regression (GPR)

The Gaussian process regression (GPR) model falls into the category of supervised machine learning methods^56^. GPR is a kernel-based non-parametric based on Bayes, with high computational efficiency and accuracy which its operation is easy for users^57^. This approach can solve classification and regression problems. This method has a high capability in modeling complex nonlinear issues^58^. A Gaussian process is expressed by the mean function $m\left( x \right)$ and the covariance function $k\left( {x_{i} ,x_{j} } \right)$ as follows^59,60^:

4 $$f\left( x \right)\sim GP\left( {m\left( x \right),k\left( {x_{i} ,x_{j} } \right)} \right).$$

In the regression problem, $y$ is defined as observations and $\varepsilon$ as noise. This noise has an average of zero and $\sigma_{n}^{2}$ variance. As a result, the Gaussian process regression model can be expressed as follows:

5 $$y = f\left( x \right) + \varepsilon , \varepsilon \sim N\left( {0,\sigma_{n}^{2} } \right).$$

In the above equation, $x$ is the input data matrix, $y$ is the output data vector, and $f$ is the values of the GPR function. The joint distribution is defined by the kernel function as follows:

6 $$\left[ {\begin{array}{*{20}c} y \\ {f_{*} } \\ \end{array} } \right]\sim N\left( {0,\left[ {\begin{array}{*{20}c} {K\left( {X,X} \right) + \sigma_{n}^{2} I_{n} } & {K\left( {x_{*} ,X} \right)^{T} } \\ {K\left( {x_{*} ,X} \right)} & {K\left( {x_{*} ,x_{*} } \right)} \\ \end{array} } \right]} \right)$$

where $K\left( {x_{*} ,X} \right)$ is equal to

7 $$K\left( {x_{*} ,X} \right) = \left[ {k\left( {x_{*} ,x_{1} } \right),k\left( {x_{*} ,x_{2} } \right), \cdots ,k\left( {x_{*} ,x_{n} } \right)} \right]$$

X is the training input matrix $X = \left[ {x_{1} ,x_{2} , \ldots ,x_{n} } \right]^{T}$, y is the training output vector $y = \left[ {y_{1} ,y_{2} , \ldots ,y_{n} } \right]^{T}$, ${\text{x}}_{*}$ is the test input vector and ${\text{f}}_{*}$ is the output for the test input data vector.

Finally, the predictor distribution is expressed by the following equation:

8 $$f_{*} |X,y,x_{*} \sim N\left( {\left( {\overline{{f_{*} }} } \right),cov\left( {f_{*} } \right)} \right)$$

where $\overline{{f_{*} }}$ and $cov\left( {f_{*} } \right)$ are defined as follows:

9 $$\overline{{f_{*} }} = K\left( {x_{*} ,X} \right)\left[ {K\left( {X,X} \right) + \sigma_{n}^{2} I_{n} } \right]^{ - 1} y$$

10 $$cov\left( {f_{*} } \right) = K\left( {x_{*} ,x_{*} } \right) - K\left( {x_{*} ,X} \right)\left[ {K\left( {X,X} \right) + \sigma_{n}^{2} I_{n} } \right]^{ - 1} K\left( {x_{*} ,X} \right)^{T} .$$

The covariance function is used to measure the effect of data points on each other^57^. This function shows the number of coordinated changes between the two variables. The proper selection of kernel function (covariance) is one of the essential factors affecting the performance of the GPR model. Numerous kernel functions are defined for use in the GPR model^59^. In the present study, ten types of kernels were examined and evaluated. Table 2 shows the kernel equations used in the present study.

**Table 2:** List of Kernel functions used for GPR model.

Kernel function	Kernel equation
Rational quadratic	$k\left( {x_{i} , x_{j} {|}\theta } \right) = \sigma_{f}^{2} \left( {1 + \frac{{r^{2} }}{{2\alpha \sigma_{l}^{2} }}} \right)^{ - \alpha }$
Exponential Kernel	$k\left( {x_{i} , x_{j} {|}\theta } \right) = \sigma_{f}^{2} exp\left[ { - \frac{r}{{\sigma_{l} }}} \right]$
Matern 3/2	$k\left( {x_{i} , x_{j} {|}\theta } \right) = \sigma_{f}^{2} \left( {1 + \frac{{\sqrt {3 } r}}{{\sigma_{l} }}} \right)exp\left[ { - \frac{{\sqrt {3 } r}}{{\sigma_{l} }}} \right]$
Squared exponential	$k\left( {x_{i} , x_{j} {|}\theta } \right) = \sigma_{f}^{2} exp\left[ { - \frac{1}{2}\frac{{\left( {x_{i} - x_{j} } \right)^{T} \left( {x_{i} - x_{j} } \right)}}{{\sigma_{l}^{2} }}} \right]$
Matern 5/2	$k\left( {x_{i} , x_{j} {|}\theta } \right) = \sigma_{f}^{2} \left( {1 + \frac{{\sqrt {5 } r}}{{\sigma_{l} }} + \frac{{5r^{2} }}{{3\sigma_{l}^{2} }}} \right)exp\left[ { - \frac{{\sqrt {5 } r}}{{\sigma_{l} }}} \right]$
ARD squared exponential	$k\left( {x_{i} , x_{j} {|}\theta } \right) = \sigma_{f}^{2} exp\left[ { - \frac{1}{2}\sum\nolimits_{m = 1}^{d} {\frac{{\left( {x_{im} - x_{jm} } \right)^{2} }}{{\sigma_{m}^{2} }}} } \right]$
ARD exponential	$k\left( {x_{i} , x_{j} {|}\theta } \right) = \sigma_{f}^{2} exp\left[ { - \varphi } \right]$
ARD matern 3/2	$k\left( {x_{i} , x_{j} {|}\theta } \right) = \sigma_{f}^{2} \left( {1 + \sqrt {3 } \varphi } \right)exp\left[ { - \sqrt {3 } \varphi } \right]$
ARD matern 5/2	$k\left( {x_{i} , x_{j} {|}\theta } \right) = \sigma_{f}^{2} \left( {1 + \sqrt {5 } \varphi + 5\varphi^{2} } \right)exp\left[ { - \sqrt {5 } \varphi } \right]$
ARD rational quadratic	$k\left( {x_{i} , x_{j} {|}\theta } \right) = \sigma_{f}^{2} \left( {1 + \frac{1}{2\alpha }\mathop \sum \limits_{m = 1}^{d} \frac{{\left( {x_{im} - x_{jm} } \right)^{2} }}{{\sigma_{m}^{2} }}} \right)^{ - \alpha }$

Where $\sigma_{f}$ is the signal standard deviation (Std), $\sigma_{l}$ is the characteristic length scale, *r* is the Euclidean distance between $x_{i}$ and $x_{j}$ which is defined by $r = \sqrt {\left( {x_{i} - x_{j} } \right)^{T} \left( {x_{i} - x_{j} } \right)}$, $\alpha$ is a positive-valued scale-mixture parameter, $\varphi = \sqrt {\sum\nolimits_{m = 1}^{d} {\frac{{\left( {x_{im} - x_{jm} } \right)^{2} }}{{\sigma_{m}^{2} }}} }$ and $\sigma_{m}$ is a separate length scale for each predictor $m$, $m = 1,2, \ldots ,d$. The values of $\theta = \left\{ {l,\sigma_{f}^{2} ,\sigma_{l}^{2} } \right\}$ (hyper-parameters) is calculated by maximizing the marginal likelihood^61,62^.

#### Kernel extreme learning machine (KELM)

ELM is a developed version of the single-layer feed-forward network (SLFN) with a random nature presented by Huang et al.^63^. The ELM consists of three layers: input, hidden, and output layers. The main advantages of this method are (1) easy implementation, (2) fast training speed, and (3) powerful generalization capability (Huang et al. 2012).

The mathematical formulation of the ELM for a dataset including M samples, by considering the number of hidden layer nodes equal to H, can be expressed as,

11 $$y_{l} = \mathop \sum \limits_{j = 1}^{H} \rho_{j} g(b_{j} x_{l} + c_{j} ),\quad l = 1,2, \ldots , M$$

where $\rho_{j}$ denotes the output weight vector, which connects the jth hidden layer node and output layer node. $g\left( x \right)$ represents the ELM activation function (AF), $b_{j}$ is the weight of the input dataset, and $c_{j}$ is the bias value for the jth hidden layer node. Equation (8) can be defined as,

12 $$Y = G\rho$$

where $Y$ denotes the model output, $G$ is the matrix of hidden layer output, which is expressed as,

13 $$G = \left[ {\begin{array}{*{20}c} {g\left( {b_{1} x_{1} + c_{1} } \right)} & \ldots & {g\left( {b_{H} x_{1} + c_{H} } \right)} \\ \vdots & \ldots & \vdots \\ {g\left( {b_{1} x_{M} + c_{1} } \right)} & \ldots & {g\left( {b_{H} x_{M} + c_{H} } \right)} \\ \end{array} } \right].$$

The ELM uses a fitness function to determine the optimum value for the $\rho$, which is given as,

14 $$F = \mathop \sum \limits_{l = 1}^{M} \left( {\mathop \sum \limits_{j = 1}^{H} { }\rho_{j} g(b_{j} x_{l} + c_{j} } \right) - T_{l} )^{2}$$

where $T$ is the target vector.

Based on the generalized inverse theory, the solution of Eq. (12) is defined as,

15 $$\rho = G^{\dag } Y$$

where $G^{\dag }$ refers to the Moore–Penrose inverse matrix (MPIM) of $G$. Regarding the orthogonal projection technique and theory of ridge regression^64^, the regularization factor (RF) was applied in the process of optimization so that the $\rho$ can be achieved as,

16 $$\rho = \left( {G^{T} G + \frac{I}{RF}} \right)^{ - 1} G^{T} Y$$

where $I$ denotes the identity matrix. Accordingly, the ELM output function is defined as,

17 $$z\left( x \right) = g\left( x \right)\rho = g\left( x \right)\left( {G^{T} G + \frac{I}{RF}} \right)^{ - 1} G^{T} Y.$$

**Figure. 3:**
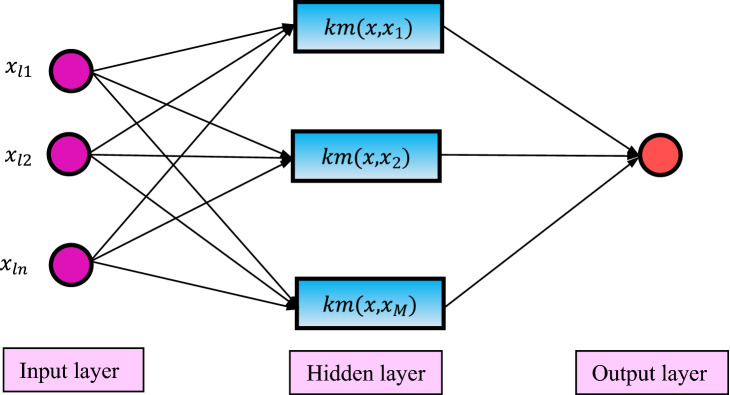
KELM structure.

Notwithstanding the suitable efficiency of the ELM, but because this method is random, it may be trapped in the local optima. Therefore, the kernel extreme learning machine (KELM) was presented by Huang et al.^65^. The main structure of the KELM is displayed in Fig. 3. In this method, a kernel matrix (KM) ($KM(x,x_{j} )$) is employed instead of the AF ($g\left( x \right)$). The KM can be formulated based on Eq. (18).

18 $$\phi = G^{T} G: \phi_{j,l} = g\left( {x_{j} } \right)g\left( {x_{l} } \right) = KM(x_{j} ,x_{l} )$$

The output function of the proposed KELM is expressed as,

19 $$f\left( x \right) = \left[ {\begin{array}{*{20}c} {KM\left( {x,x_{1} } \right)} \\ \vdots \\ {KM\left( {x,x_{H} } \right)} \\ \end{array} } \right]\left( {\phi + \frac{I}{RF}} \right)^{ - 1} Y.$$

In this work, the radial basis function (RBF) is utilized as a KM, which can be obtained as,

20 $$KM\left( {x_{j} ,x_{l} } \right) = {\text{exp}}\left( { - \frac{{\left| {\left| {x_{j} - x_{l} } \right|} \right|^{2} }}{\mu }} \right)$$

where $\mu$ is a constant number.

#### Generalization regression neural network (GRNN)

Generalized regression neural network (GRNN) is a kind of radial basis function network (RBFN) that is based on kernel regression^66^. Unlike the conventional neural networks (CNN), the GRNN does not need a repetitive training process like the back-propagation technique. The GRNN does not stick to local solutions^67–69^. This method comprises four layers: input, pattern, summation, and output layers.

The input layer receives the input dataset ($x$). In this layer, the number of neuros is equal to the dimension of the input dataset. In the pattern layer, neurons using a nonlinear function transform the input dataset ($x$) to $p_{k}$ (i.e., the output of the pattern layer) based on the following equation:

21 $$p_{k} = \exp \left[ {\frac{{\left( {x - x_{k} } \right)^{T} \left( {x - x_{k} } \right)}}{{2\rho^{2} }}} \right]$$

where x_k_ denotes the training sample of the *k*th neuron in the input layer. $\rho$ is the spread factor.

The third layer (i.e., summation layer) consists of two types of neurons: (1) one simple neuron and (2) *m* weighted neurons, which are specified by $S_{o}$ and $S_{t}$. These kinds of neurons are defined as,

22 $$S_{o} = \mathop \sum \limits_{k = 1}^{M} p_{k} ,\quad S_{t} = \mathop \sum \limits_{k = 1}^{M} y_{k} p_{k}$$

where $y_{k}$ is the target dataset.

The output layer (i.e., output layer) divides the summation layer results to achieve the output predicted result, which is expressed as,

23 $$Y = \frac{{S_{t} }}{{S_{o} }}.$$

#### Surface response methodology (RSM)

In the present study, RSM was used to investigate the effect of independent variables (geometric and hydraulic conditions) on the output (response) variable (side orifice discharge coefficient) and also to provide an optimal regression relationship for the elliptical side orifice discharge coefficient prediction. The RSM method is a statistical tool for modeling and analyzing the behavior of the process (input) variables on the response (output) variable^70^. Using RSM, most information can be obtained with a minimum of experimental data. The 2nd order RSM model includes linear, quadratic, and the interaction of input variables sentences. The RSM model for the above case can be expressed as follows^71,72^:

24 $$y = \mathop \sum \limits_{i = 1}^{k} \alpha_{i} X_{i} + \mathop \sum \limits_{i = 1}^{k} \alpha_{ii} X_{i}^{2} + \mathop \sum \limits_{i = 1}^{k - 1} \mathop \sum \limits_{j = i + 1}^{k} \alpha_{ij} X_{i} X_{j} + \varepsilon$$

where $X$ is the input data matrix, $y$ is the output data estimation vector, $\varepsilon$ is a random error vector and $\alpha_{i} ,\alpha_{ii} \;and\;\alpha_{ij}$ are regression coefficients which the following equation can calculate:

25 $$\alpha = \left( {X^{T} X} \right)^{ - 1} X^{T} Y$$

**Figure 4:**
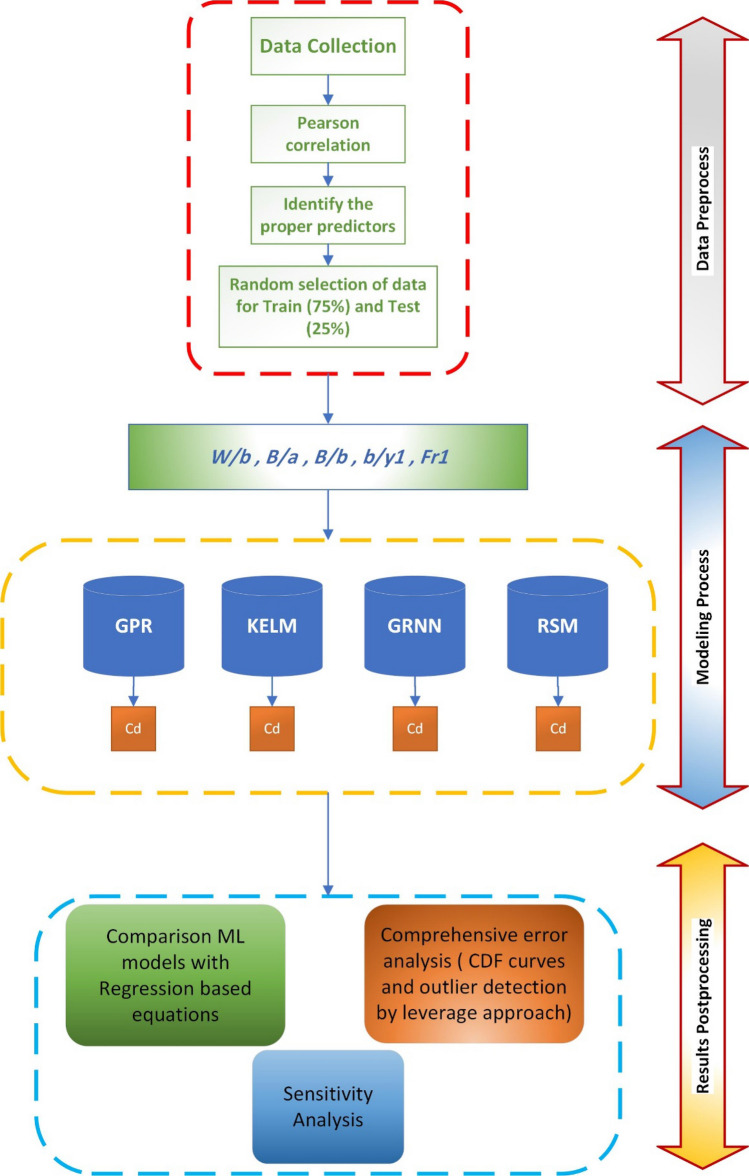
The flowchart of predicting discharge coefficient of elliptical side orifice by different machine learning (ML) models.

A flowchart of the machine learning models for the discharge coefficients of the elliptical side weir can be depicted in Fig. 4. In all models, the input is normalized using the following formula:

26 $$x_{nor} = \frac{{x - x_{min} }}{{x_{max} - x_{min} }}$$

where $x$ is the value of variable and $x_{min}$ and $x_{max}$ are the minimum and maximum value of the variable, respectively.

### Accuracy criteria of approaches

Five statistical indices evaluated the models: root mean square error (RMSE), mean average percentage error (MAPE), correlation coefficient (R), normalized root mean square error (NRMSE), and mean bias error (MBE). The relationships of each of the mentioned parameters are presented below:

27 $$R = \frac{{\mathop \sum \nolimits_{i = 1}^{N} (Cdo_{i} - \overline{Cdo} )\left( {Cdp_{i} - \overline{Cdp} } \right)}}{{\sqrt {\mathop \sum \nolimits_{i = 1}^{N} (Cdo_{i} - \overline{Cdo} )^{2} \mathop \sum \nolimits_{i = 1}^{N} (Cdp_{i} - \overline{Cdp} )^{2} } }}$$

28 $$RMSE = \sqrt {\frac{{\mathop \sum \nolimits_{i = 1}^{N} (Cdo_{i} - Cdp_{i} )^{2} }}{N}}$$

29 $$MBE = \frac{1}{N}\mathop \sum \limits_{i = 1}^{N} \left( {Cdo_{i} - Cdp_{i} } \right)$$

30 $$NRMSE = \frac{RMSE}{{\overline{Cdo} }}$$

31 $$MAPE = \frac{1}{N}\mathop \sum \limits_{i = 1}^{N} \left| {\frac{{Cdo_{i} - Cdp_{i} }}{{Cdo_{i} }}} \right| \times 100.$$

At the above equations, $Cdo_{i}$ and $Cdp_{i}$ respectively are observed and predicted values of discharge coefficient of elliptical side orifice, $\overline{Cdo}$ is the mean value of observations, $\overline{Cdp}$ is the mean value of predictions, and N is the number of data.

### Outlier detection with leverage approach

Through developing a mathematical model, it is necessary to detect outlier data obtained from the model. Several methods have been proposed to identify and detect outlier data. Among these, the leverage approach is one of the most well-established and widely used approaches. In this method, the difference between the actual data and the data obtained from the model is defined as the residual. To calculate the leverage index ($hat$) the following matrix must be calculated:

32 $$H = X\left( {X^{T} X} \right)X^{ - 1} .$$

In the above equation, $X$ is an $n \times k$ matrix, where $n$ is the number of samples and $k$ is the number of model variables. The diagonal elements of the $H$ matrix are known as the hat or leverage index. The standard residual percent ($R$) is plotted against the $hat$ to indicate the domain of application of the model and the outlier data. The warning value of the leverage $H_{*}$ is calculated from the following equation:

33 $$H_{*} = 3 \left( {k + 1} \right) / n.$$

The plot of $R$ versus $hat$ is known as the Williams diagram. If most of the data are in the range of $- 3 < R < 3$ and $0 < H < H_{*}$ It indicates the appropriate application of the model in the mentioned range and, therefore, shows the developed model's statistical validity^73,74^.

## Results and discussion

This section discusses and evaluates the results obtained from GPR, KELM, GRNN, and RSM models and regression-based models. There will also be a comprehensive comparison between the mentioned AI models and regression-based models. Error analysis was performed using CDF curves, relative error, and leverage approach. Finally, sensitivity analysis will be performed to determine the parameters affecting the elliptical side orifice discharge coefficient. All models are performed in the MATLAB 2020a software on a personal computer (Intel Core i7 2.6 GHz processor and 16 GB RAM).

### Gaussian process regression (GPR) model

**Figure 5:**
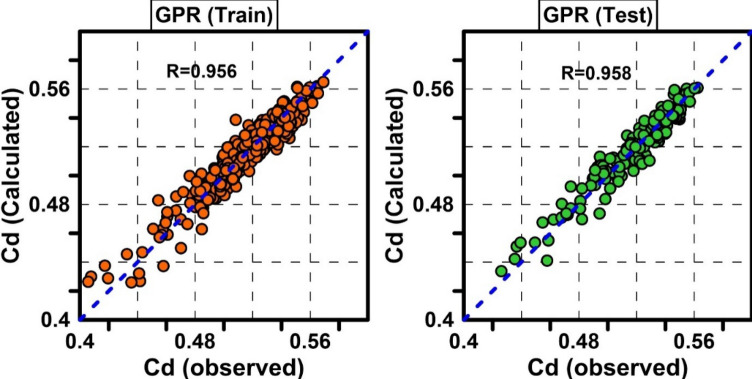
Scatter plots of observed Cd against predicted Cd by GPR model for train and test data.

The GPR model was created using the dimensionless variables mentioned in the previous section as input and the discharge coefficient ($Cd$) parameter as output. The most important factor in the performance of the GPR model is the type of kernel and its parameters. In the present study, ten kernels and LBFGS-based quasi-Newton methods were used to optimize kernel parameters. Table 3 shows the results obtained from different kernels with their optimal parameter. The results obtained by different kernels were compared using R and RMSE statistical parameters for the test data series. According to Table 3, the *ARDsquaredexponential* kernel with R = 0.9579, RMSE = 0.0081 and MAPE = 1.3243% had the best performance in estimating the orifice discharge coefficient. The *ARDMatern* 5/2 kernel with R = 0.9571, RMSE = 0.0087 and MAPE = 1.5782% was the second model with high accuracy. The weakest performance was provided by *exponential* kernel with R = 0.9509, RMSE = 0.0087 and MAPE error percentage = 1.4063%. The results of the optimal GPR model for the training and test data series are presented in Fig. 5.

**Table 3:** Effect of GPR kernel type on model accuracy.

Kernel	RMSE	R	MAPE	MBE	NRMSE
Exponential	0.00875	0.95098	1.40634	− 0.00062	1.69007
Squaredexponential	0.00845	0.95423	1.36432	− 0.00054	1.63369
Matern3/2	0.00857	0.95299	1.38097	− 0.00061	1.65591
Matern5/2	0.00853	0.95346	1.37472	− 0.00059	1.64759
Rational quadratic	0.00845	0.95423	1.36432	− 0.00054	1.63370
ARDexponential	0.00840	0.95474	1.38167	− 0.00031	1.62279
ARDsquaredexponential	0.00810	0.95797	1.32428	− 0.00029	1.56469
ARDmatern3/2	0.00821	0.95674	1.35327	− 0.00028	1.58723
ARDmatern5/2	0.00817	0.95723	1.34383	− 0.00028	1.57823
ARDrationalquadratic	0.00818	0.95715	1.34447	− 0.00028	1.57975

### Kernel extreme learning machine (KELM)

**Figure 6:**
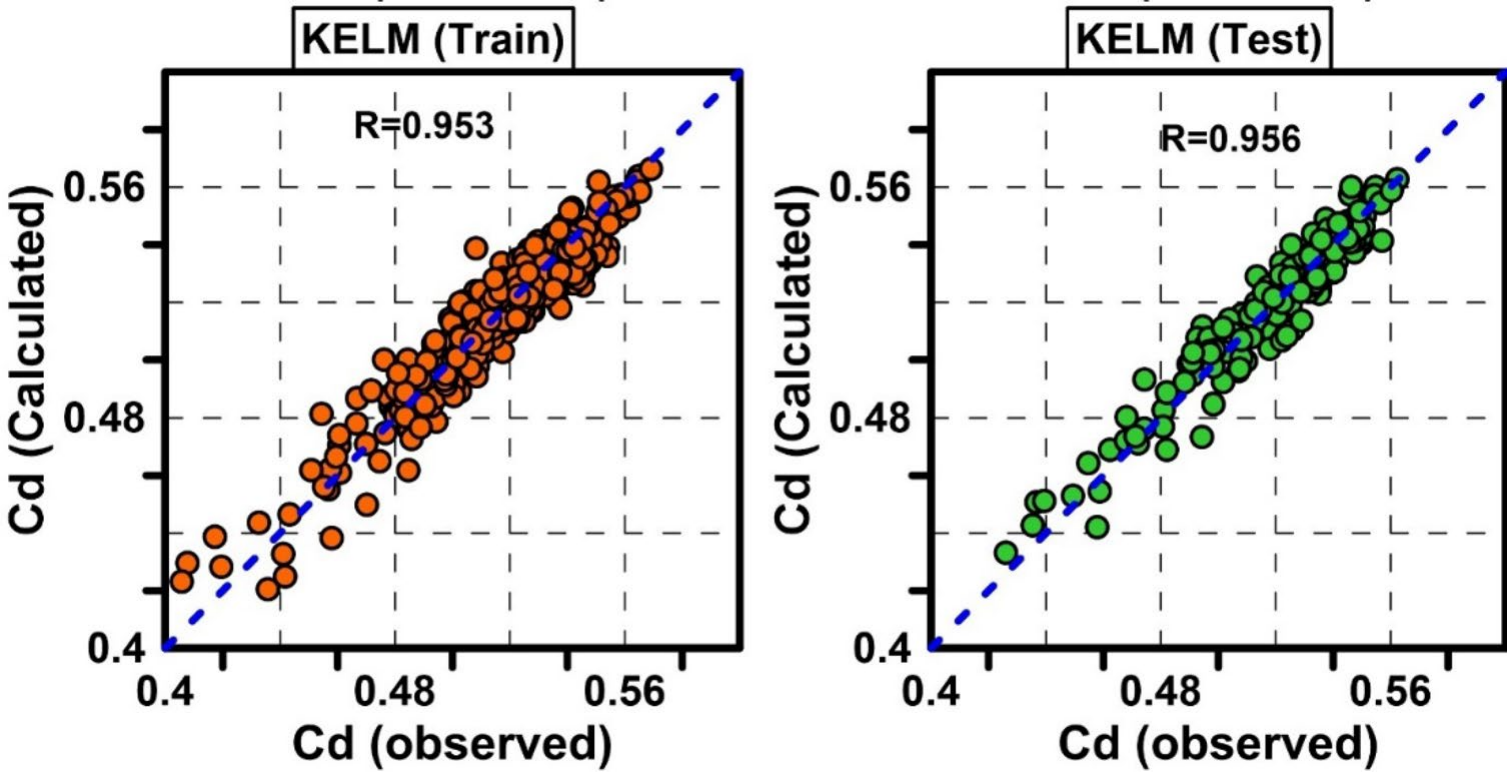
Scatter plots of observed Cd against predicted Cd by KELM model for train and test data.

In the KELM model, the RBF kernel was considered as the model kernel^75,76^. The RBF kernel has one parameter as $\sigma ,$ and the KELM model has one parameter as an adjustment parameter ($C$). The grid serach method was used to obtain $\sigma$ and $C$. The values of $\sigma$ and $C$ were changed from 0.01 to 3 and 1 to 1000, respectively. Finally, the optimal values of $\sigma = 0.1$ and C = 600 were obtained for the test data series. According to the optimal parameters of the kernel and KELM model, the best model was obtained with R = 0.9564, RMSE = 0.0082, and MAPE = 1.3499%. The results of the optimal KELM model are presented in Fig. 6 for the test and training datasets.

### Generalized regression neural network (GRNN)

**Figure 7:**
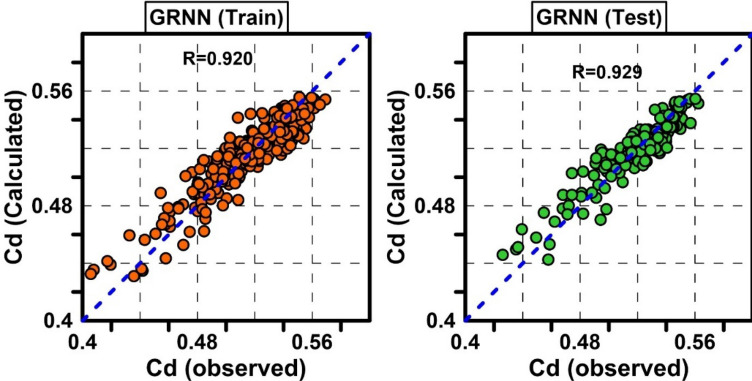
Scatter plots of observed Cd against predicted Cd by GRNN model for train and test data.

The only parameter in the GRNN model is the Spread parameter^77^. To obtain the optimal spread value, its values were changed between 0.01 and 10 with 0.01 intervals. The results showed that the optimal value of this parameter is 0.05. R = 0.929, RMSE = 0.0106 and MAPE = 1.6971% were obtained for the optimal GRNN model. The results of the optimal GRNN model are presented in Fig. 7 for the test and training datasets.

### Response surface methodology (RSM)

The effect of independent variables on the side elliptical orifice discharge coefficient was evaluated using the RSM model. One of the advantages of the RSM is presenting a regression relationship between input and output variables. The RSM model is based on the number of independent variables, their squares, and their relationship (in pairs). The equation obtained from this model is as follows:

34 $$\begin{aligned} Cd & = 0.0717 + 0.1817W/b + 0.1398B/a - 0.0077B/b + 3.9383b/y1 \\ & \quad - \;0.2686Fr1 - 0.0234W/b \cdot B/a - 0.0074W/b \cdot B/b - 0.6560W/b \cdot b/y1 \\ & \quad - \;0.0087B/a \cdot B/b - 1.3551B/a \cdot b/y1 - 0.0411B/b \cdot Fr1 \\ & \quad - \;3.3842b/y1 \cdot Fr1 + 0.0012(B/b)^{2} + 0.6557\left( {Fr1} \right)^{2} . \\ \end{aligned}$$

**Figure 8:**
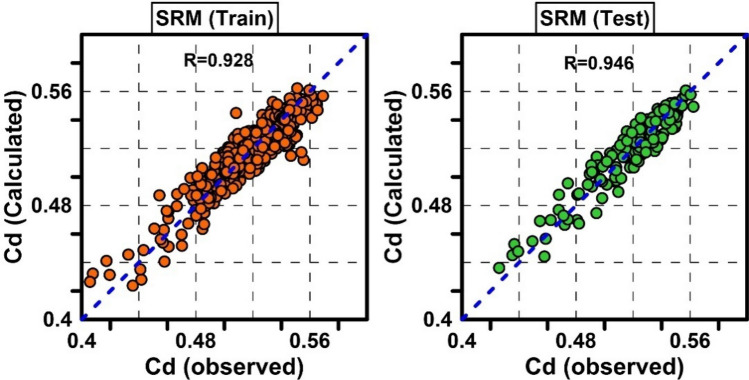
Scatter plots of observed Cd against predicted Cd by MLRI model for train and test data.

Table 4 shows the ANOVA analysis of variance for the equation and its coefficients. According to this table, all coefficients are significant (*p* value < 0.05). In the RSM model, R = 0.9456, RMSE = 0.0092 and MAPE = 1.4921% were obtained for the test dataset. Figure 8 shows the performance of the RSM model for training and test data.

**Table 4:** ANOVA results for determining the effective variable interactions in the RSM model.

Variables	Coefficient	Sum of squares	F-value	*p* value
$W/b$	0.18167	0.06822	695.09123	0.00000
$B/a$	0.13982	0.09677	986.01148	0.00000
$B/b$	0.00772	0.00170	17.35857	0.00004
$b/y1$	3.93829	0.00163	16.65797	0.00005
$Fr1$	− 0.26858	0.03962	403.65941	0.00000
$W/b \times B/a$	− 0.02340	0.00205	20.87685	0.00001
$W/b \times B/b$	− 0.00743	0.00371	37.77965	0.00000
$W/b \times b/y1$	− 0.65597	0.00503	51.25667	0.00000
$B/a \times B/b$	− 0.00870	0.00078	7.95540	0.00502
$B/a \times b/y1$	− 1.35512	0.00152	15.45248	0.00010
$B/a \times Fr1$	0.26254	0.00127	12.95195	0.00036
$B/b \times b/y1$	0.11749	0.00199	20.24286	0.00001
$B/b \times Fr1$	− 0.04114	0.00197	20.08285	0.00001
$b/y1 \times Fr1$	− 3.38425	0.00159	16.21657	0.00007
$(B/b)^{2}$	0.00118	0.00128	13.02753	0.00034
$\left( {Fr1} \right)^{2}$	0.65565	0.00319	32.46088	0.00000

### Regression-based equations

Vatankhah and Rafeifar^3^ presented five regression-based models to calculate the elliptical side orifice discharge coefficient.

35 $$Equation\;1:C_{d} = 0.64 - 0.1\left( \frac{W}{b} \right)^{0.572} \left( \frac{B}{a} \right)^{1.27} \left( \frac{B}{b} \right)^{0.04} \left( \frac{b}{y1} \right)^{0.13} Fr1^{0.85}$$

36 $$Equation\;2:C_{d} = 0.635 - 0.085\left( \frac{W}{b} \right)^{0.57} \left( \frac{B}{a} \right)^{1.33} Fr1^{0.91}$$

37 $$Equation\;3:C_{d} = 0.536 - 0.005\left( \frac{W}{b} \right)^{2.11} Fr1^{1.35}$$

38 $$Equation\;4:C_{d} = 0.578 - 0.039\left( \frac{B}{a} \right)^{1.47} Fr1^{0.0.24}$$

39 $$Equation\;5:C_{d} = 0.549 - 0.004\left( \frac{W}{b} \right)^{0.969} \left( \frac{B}{a} \right)^{2.631}$$

**Figure 9:**
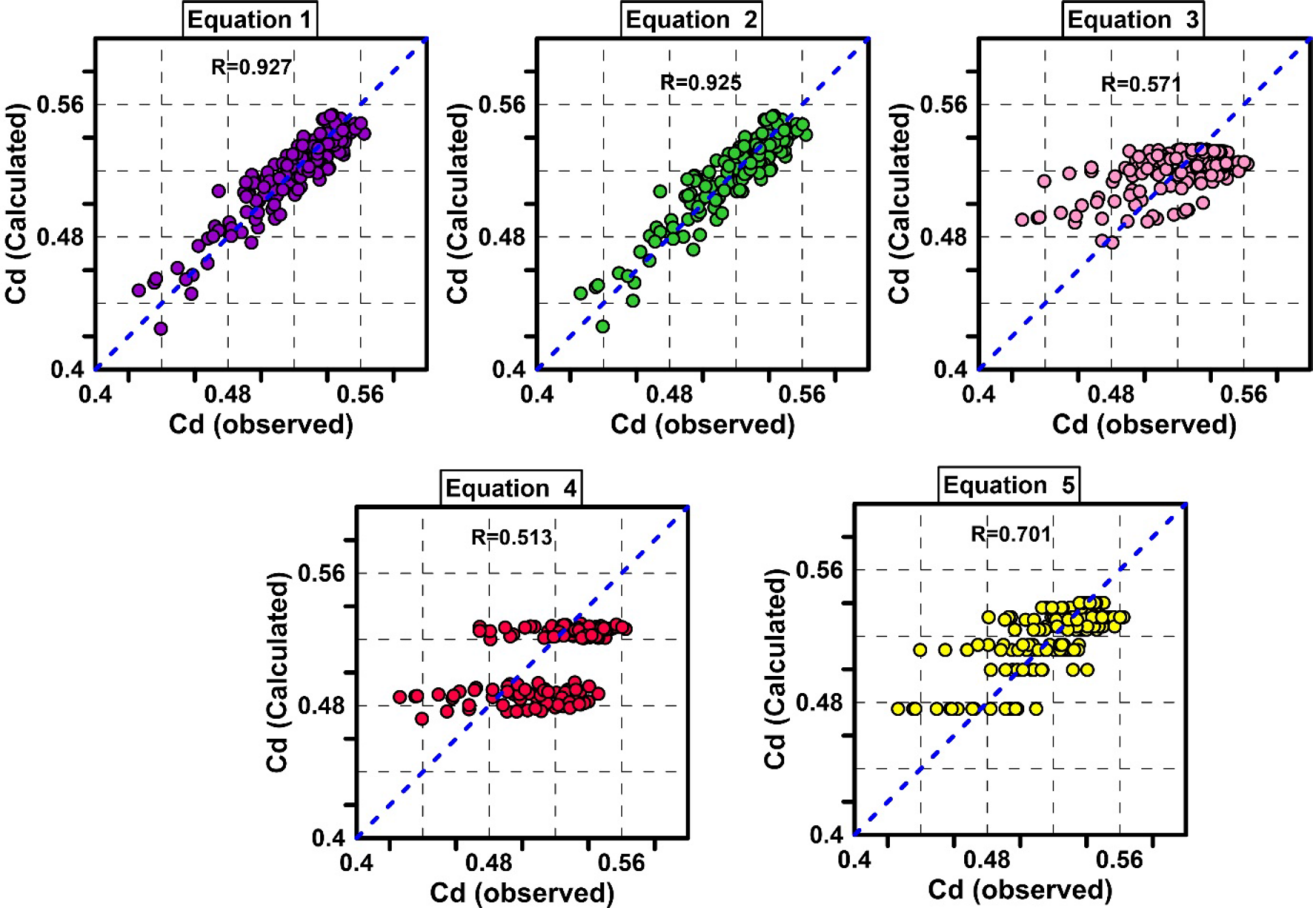
Scatter plots of regression-based equations.

Table 5 shows the results obtained from these five regression-based models. According to Table 5, Eq. (1) in which all effective parameters are involved with R = 0.9277, RMSE = 0.0106 and MAPE = 1.6846% had the best performance. Equation 2 takes into account the parameters $Fr1$, $B / a$ and $w / b$ as input with R = 0.9254, RMSE = 0.0107 and MAPE = 1.6993% is in the second rank. In equations, 3 to 5, which consider the parameters $Fr1 - w / b, Fr1 - B / a$ and $w / b - B / a$ as input, respectively, the accuracy of the equations is not acceptable, and the value of R is $R \le 0.7$. The MAPE error in Eqs. (3) to (5) models is more than 3%. Figure 9 shows the scatter plots of experimental data and regression-based equation. As shown in the figure, Eqs. (3) to (5) did not provide good results.

**Table 5:** Results of regression-based equations.

Equations	RMSE	R	MAPE	MBE	NRMSE
Equation 1	0.0106	0.9277	1.6846	0.0003	2.0464
Equation 2	0.0107	0.9254	1.6993	0.0007	2.0731
Equation 3	0.0234	0.5713	3.6714	− 0.0014	4.5212
Equation 4	0.0273	0.5149	4.3799	0.0113	5.2789
Equation 5	0.0202	0.7008	3.1016	− 0.0011	3.9039

### Comparison between models

GPR, KELM, GRNN, and RSM models were developed to predict the side elliptic orifice discharge coefficient in the previous section, and their optimal parameters were obtained. This section will compare the machine learning models developed in the previous section and the top regression model. Table 6 shows the statistical parameters of the best results obtained from the machine learning models and the best regression model for the training and test datasets. According to Table 6, all machine learning models performed better than the regression-based model. Comparison between machine learning models also shows that the GPR model, with R = 0.9556, RMSE = 0.0077 for training data, and R = 0.9580 and RMSE = 0.0081 for test data, had the highest accuracy in estimating the orifice discharge coefficient. The KELM model is in the second rank with a slight difference (R = 0.953 and RMSE = 0.0080 for training data and R = 0.9564 and RMSE = 0.0082 for test data). The GRNN model had the lowest accuracy among machine learning models (R = 0.9202 and RMSE = 0.0104 for training data and R = 0.9291 and RMSE = 0.0106 for test data). The RSM model also had a good accuracy in estimating the elliptical side orifice (R = 0.9279 and RMSE = 0.0097 for training data and R = 0.9456 and RMSE = 0.0092 for test data) by presenting a regression relationship.

**Table 6:** Performance of AI models and best Vatankhah and Rafeifar^3^ regression-based equation in predicting discharge coefficient.

Statistical criteria	Models				
	GPR	GRNN	K-ELM	RSM	Equation 1
**Training stage**					
R	0.9556	0.9202	0.9530	0.9279	0.8962
RMSE	0.0077	0.0104	0.0079	0.0097	0.0116
MAPE	1.1781	1.5769	1.2128	1.4790	1.7496
MBE	0	0.0001	0	0	0.0005
NRMSE	1.4844	2.0043	1.5230	1.8742	2.2231
AVE rank	1	4	2	3	5
**Testing stage**					
R	0.9580	0.9291	0.9564	0.9456	0.9277
RMSE	0.0081	0.0106	0.0082	0.0092	0.0106
MAPE	1.3243	1.6971	1.3499	1.4921	1.6846
MBE	− 0.0003	− 0.0004	− 0.0002	− 0.0001	0.0003
NRMSE	1.5647	2.0532	1.5929	1.7738	2.0464
AVE rank	1	4	2	3	5

**Figure 10:**
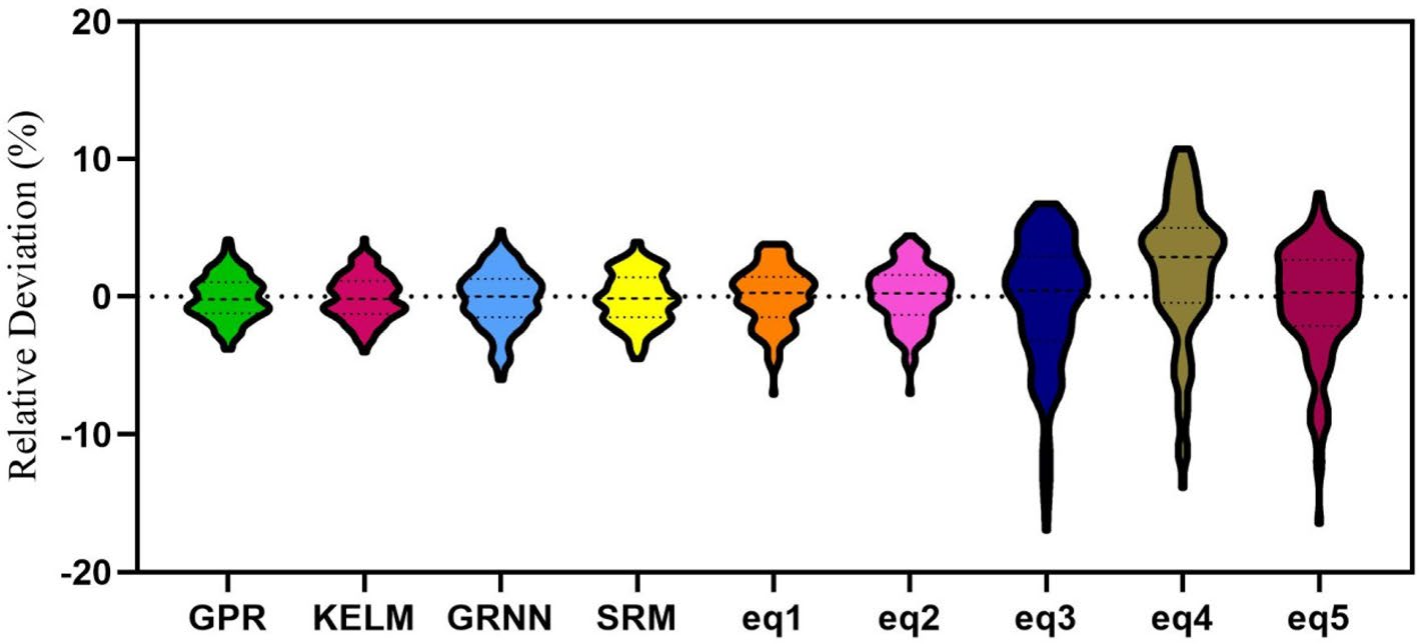
The error distribution of four developed AI models and regression-based equations.

Figure 10 shows the error distribution in a violin graph for machine learning models and five regression equations studied in the present study. According to the figure, the lowest error range is related to the GPR model [− 3.78% to + 4.146%]. After the GPR model, the KELM model is in second place with an error range [− 3.981% to + 4.222%]. The GRNN model with the error range [− 5.99% to + 4.833%] has the highest error range among machine learning models. According to Fig. 10, regression-based models have more error ranges. The best regression model (Eq. 1) has an error range of [− 7.057% to + 3.835%]. Equations (3) to (5) have the largest error range.

**Figure 11:**
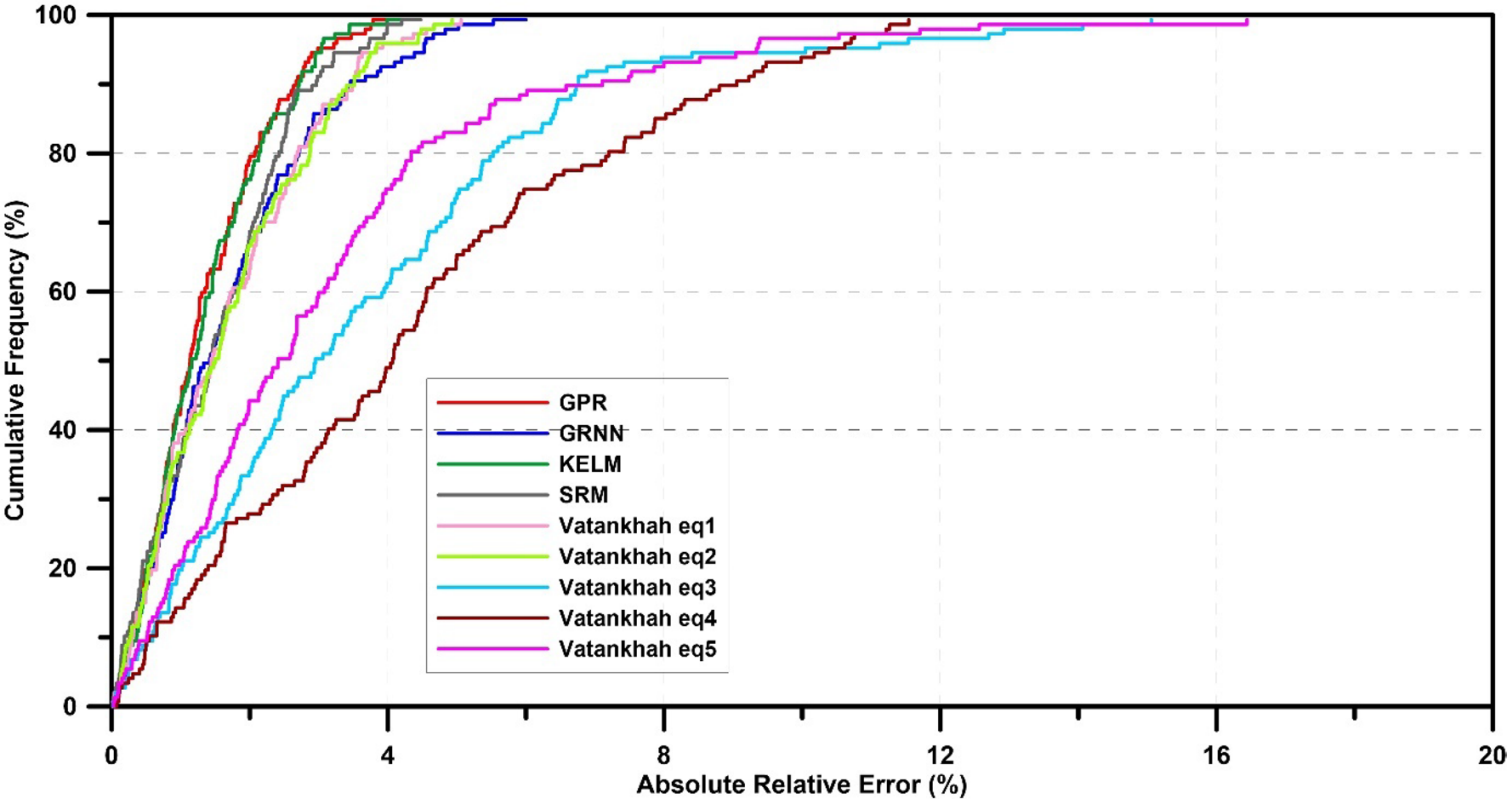
The cumulative frequency (%) of absolute relative error (%) for AI models and regression-based equations.

Figure 11 shows the cumulative frequency versus absolute error percentage. According to Fig. 11, the GPR model provides an error of less than 1.7% for 70% of the data. This number is 1.74% for the KELM model and 2.16 and 2.03% for GRNN and RSM models. As a result, the GPR model is more accurate and reliable in estimating the elliptical side orifice discharge coefficient. In regression models, Eqs. (1) and (2) for 70% of the data represent an absolute error percentage of less than 2.2%. In Eqs. (3) to (5), the values of this number are equal to 4.76%, 5.7%, and 3.65%, respectively. The mentioned results of the analysis of the cumulative frequency curve against the absolute percentage of error show the superiority of machine learning models over regression-based models.

**Figure 12:**
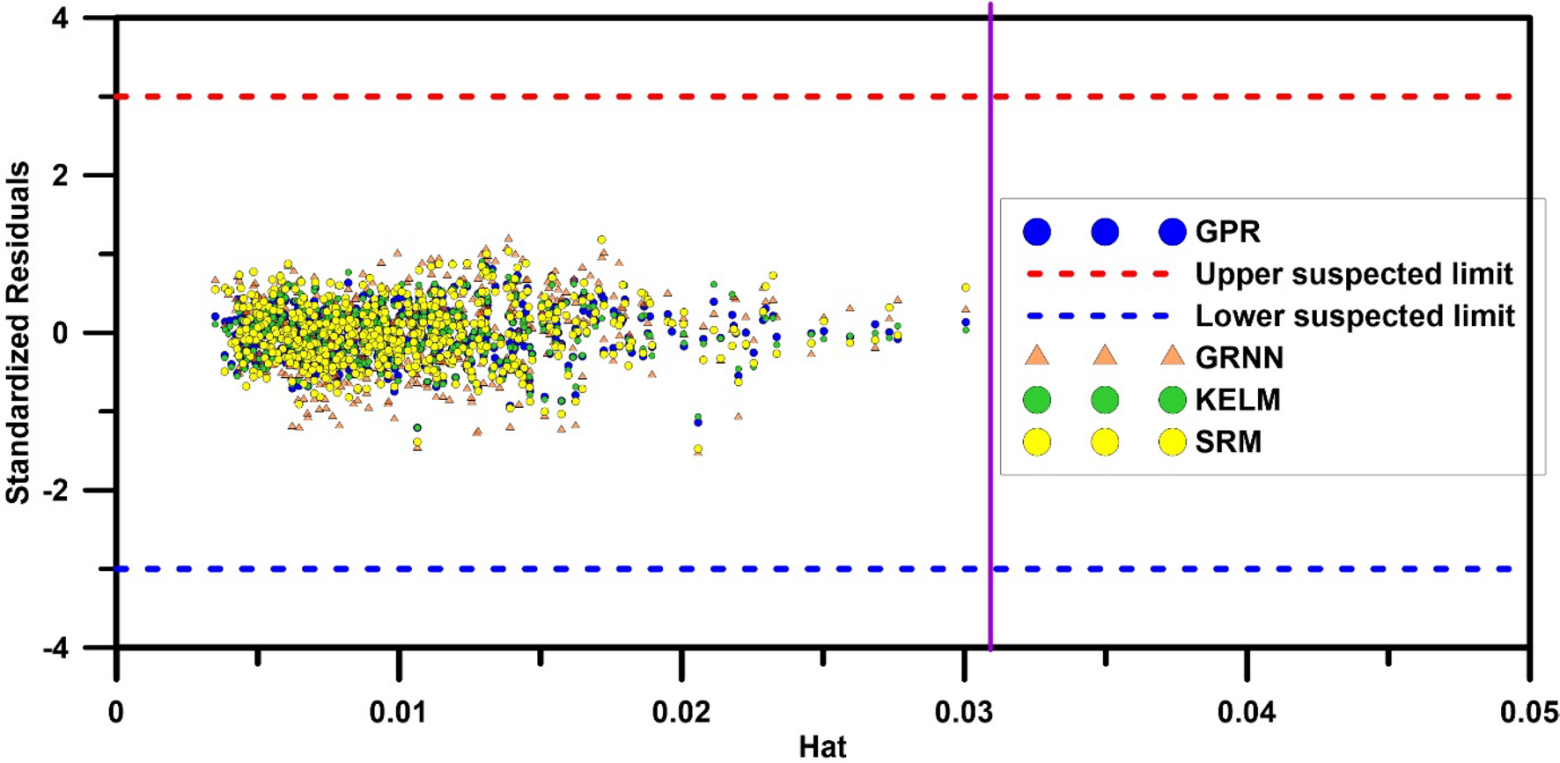
Williams plot to identify the application domain of machine learning models.

**Figure 13:**
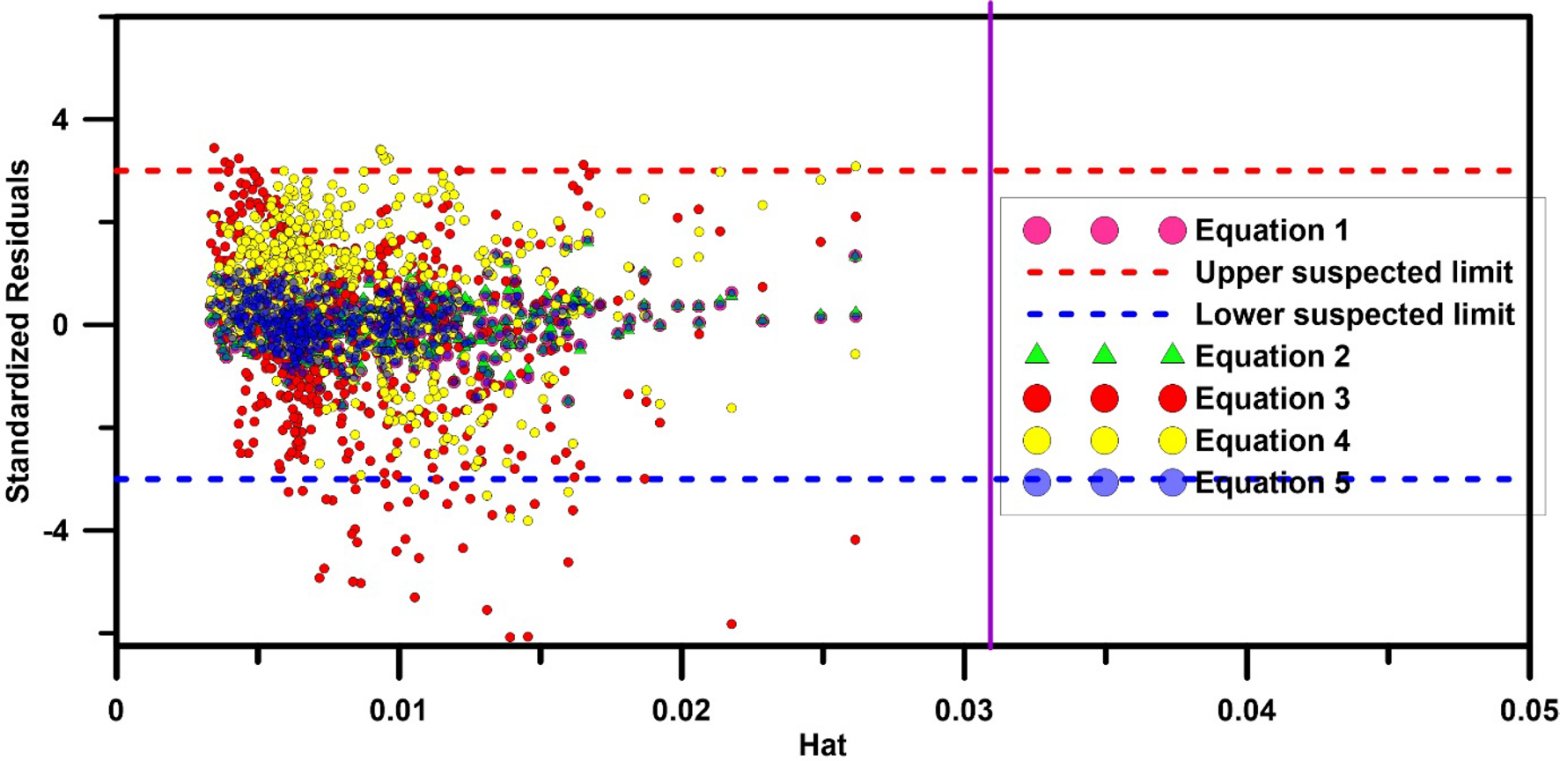
Williams plot for identifying the application domain of regression-based models.

Finally, to ensure the statistical validity of the developed models, the values of $H$ matrix, leverage index (hat), standard residual percentage $R$ and warning value of leverage $H_{*}$ was calculated according to the leverage approach, and the Williams diagram was plotted for all machine learning and regression-based models. Figure 12 shows the Williams diagram for the GPR, GRNN, KELM, and RSM machine learning models. According to Fig. 12 in all models, the data obtained from the models are in the range of $- 3 < R < 3$ and $0 < H < H_{*}$ And are therefore statistically valid. Figure 13 shows the Williams diagram for regression-based models. As can be seen from Fig. 13, Eqs. (1), (2), and (5) are statistically valid and are in the range of $- 3 < R < 3$ and $0 < H < H_{*}$ But the Eqs. (3) and (4) are not in the range of confidence, and therefore their application is not recommended in estimating the discharge coefficient of elliptical side orifice.

### Sensitivity analysis

A sensitivity analysis was performed on the data using the GPR model (superior model) to determine the variables affecting the elliptical side orifice discharge coefficient. One of the reliable methods in sensitivity analysis is omitting each data variable and determining statistical parameters in the absence of this variable in model^78^. Table 7 shows the sensitivity analysis results of the variables affecting the elliptical side orifice discharge coefficient. According to Table 7, omitting the parameter $B / a$ (channel width to orifice length) had the greatest effect on reducing the model accuracy (R = 0.7932). Therefore $B / a$ is the most effective parameter in determining the elliptical side orifice discharge coefficient. The Froude number ($Fr1$) with R = 0.8968 is the second parameter affecting the discharge coefficient. The parameters $w / b$ with R = 0.9052, $b / y1$ with R = 0.9432 and $B / b$ with R = 0.9576 are in the rank of 3 to 5 parameters affecting the discharge coefficient.

**Table 7:** The statistical measures for sensitivity analysis situations.

Metrics	All-$W/b$	All-$B/a$	All-$B/b$	All-$b/y1$	All-$Fr1$	All
R	0.9052	0.7932	0.9576	0.9432	0.8968	0.9580
RMSE	0.0120	0.0174	0.0081	0.0094	0.0125	0.0081
MAPE	1.7022	2.4944	1.3336	1.4860	1.9147	1.3243
MBE	− 0.0003	− 0.0012	− 0.0003	− 0.0007	− 0.0007	− 0.0003
NRMSE	2.3179	3.3595	1.5723	1.8159	2.4168	1.5647
AVE rank	3	1	5	4	2	–

## Conclusion

In the present study, four machine learning methods KELM, GPR, GRNN, and RSM, were used to estimate the elliptical side orifice discharge coefficient. The results were compared with the proposed regression-based equations. The data used to develop the models included 588 series of laboratory data. Five dimensionless parameters: orifice crest height to orifice height ratio ($W/b$), main channel width to orifice length ratio ($B/a$), main channel width to orifice height ratio ($B/b$), upstream orifice depth (y1) to orifice height ratio (y1/b) and upstream orifice Froude number (Fr1) as the model input and the discharge coefficient of side elliptical orifice ($Cd$) were considered as model output. The results obtained from the statistical parameters of the test dataset showed that all four machine learning models had performed well in estimating the elliptical side orifice discharge coefficient, and the R-value varies between 0.9580 for the GPR model (the strongest model) to 0.9291 for the GRNN model (the weakest model). Comparing machine learning models and regression-based models showed the superiority of artificial intelligence models in estimating the orifice discharge coefficient. The highest accuracy belongs to GPR (RMSE = 0.0081, R = 0.958, MAPE = 1.3242) and KELM (RMSE = 0.0082, R = 0.9564, MAPE = 1.3499) models. The RSM model had good accuracy and provided a functional regression equation for calculating the discharge coefficient. Error analysis using cumulative error distribution curves and relative error distribution function also shows the superiority of the GPR model over other methods used in the present study. Using the RSM model, this study developed a new practical regression equation to predict the elliptic side orifice's discharge coefficient. The leveraged approach was applied to detect outliers and the model applicability domain. Results showed that all proposed machine learning models are statistically valid. Also, the sensitivity analysis result of model input parameters showed that the ($B/a$) parameter has the most impact on model performance and the ($B/b$) parameter has the least impact on model performance. The present study results can be used to refine the delivered flow measurement for optimal management of water consumption by the elliptical side orifice structure.

### Limitations and future scope

The results of this research are valid for the range of data used, and it is most used in a variety of elliptical sharp-crested side orifices. Therefore, to calculate the discharge coefficient related to different types of circular sections, more effort is needed to collect data sets related to them. The future scope can be investigated by providing an individual model capable of estimating the discharge coefficient of both circular and elliptical orifice by combining corresponded experimental data sets. Also, developing an ensemble model for integrating the advantage of each developed standalone model could be effective in enhancing the accuracy of discharge coefficient computation.

## Data Availability

The used dataset and codes in this research are available upon reasonable request from the corresponding author.
